# Effect of Preload on the Weld Quality of Ultrasonic Welded Carbon-Fiber-Reinforced Nylon 6 Composite

**DOI:** 10.3390/polym14132650

**Published:** 2022-06-29

**Authors:** Zengguo Tian, Qian Zhi, Xiangyu Feng, Guopeng Zhang, Yafei Li, Zhongxia Liu

**Affiliations:** 1 School of Physics and Microelectronics, Zhengzhou University, Zhengzhou 450001, China; tianzg@zzu.edu.cn (Z.T.); fengxiangyu_2022@163.com (X.F.); gpzhang@zzu.edu.cn (G.Z.); yflzzu@163.com (Y.L.); 2 School of Materials Science and Engineering, Hunan University of Science and Technology, Xiangtan 411201, China; 3 School of Mechanical Engineering, Shanghai Jiao Tong University, Shanghai 200240, China

**Keywords:** ultrasonic welding, carbon-fiber-reinforced nylon 6 composite, preload, poor contact, joint strength

## Abstract

Ultrasonic welding (UW) of polymeric composites is significant in automobile industry; however, maintaining the perfect contact condition between workpieces is a great concern. In this study, effect of preloading and welding pressure on strengths of UWed 2.3-mm-thick short carbon fiber reinforced nylon6 (C_f_/PA6) joints with poor contact between workpieces was investigated through stress simulation and energy dissipation at the faying interface. Results showed the application of preloading can increase the strength of normal joint by 18.7% under optimal welding parameters. Gaps between upper and lower workpieces decreased the joint strength significantly, especially for gaps greater than 1.5 mm. Preloading improved the strengths of the joints with gaps remarkably, where the strength of joints with 1.5 mm gap recovered to 95.5% of that the normal joint. When combining the weld nugget evolution, stress-deformation simulation during UW, and ultrasonic vibration transmission analysis, the improvement mechanism of the joint under preloading was mainly because the preloading compacted the contact between workpieces, which favored the energy transmission at faying interface.

## 1. Introduction

The rapid development of automobile industry has caused increasingly serious environmental pollution. To reduce fuel consumption and automobile exhaust emissions, automobile manufacturers are actively exploring lightweight and high-performance materials, such as new aluminum/magnesium alloys, and thermoplastic composites, to replace traditional automobile steel [1,2,3]. In particular, carbon-fiber-reinforced nylon-matrix composites are now widely used in mass manufacturing of automobile parts, due to their low cost, relatively high specific strength, and excellent lightweighting effect, which can reduce the weight of automobile parts by more than 40% [4,5,6,7,8].

The main challenge for the application of nylon composites in manufacturing automobile parts is solid joining of the composites. At present, the available joining techniques for thermoplastic composites include mechanical fastening, adhesive bonding and welding [9,10]. Ultrasonic welding (UW) technology is widely used because it is fast, energy efficient, suitable for mass production, and offers good cosmetic quality [11,12].

Quality of UWed joint can be affected by many factors, such as material properties, welding parameters, contact condition among horn, welding part and fixture [13,14,15]. Our previous studies [16,17,18] have systematically investigated the influence of imperfect contacts, such as horn misalignment, gaps between workpiece, and fixture, on the weld quality of UWed joints. Experimental results showed the imperfect conditions affect the joint strength severely and produce discrepant welds [18,19,20,21]. Although these discrepant joints can be repaired by application of the secondary ultrasonic pulse and other processes [22,23], the additional pulse is time-consuming and the cosmetic quality of joints is deteriorated. It is known that the ultrasonic energy transmission during UW is closely related to the contact condition among the horn, upper workpiece and lower adherend. Increasing the welding pressure can improve the contact condition at the faying surface; however, higher static pressure applied to the workpieces increases the damping of the ultrasonic vibration system and decreases the vibration amplitude, which degrades UW process control and would reduce the weld quality accordingly [24]. In addition, if the gap between upper and lower workpieces is large, then the thermoplastic composite would melt significantly or even vaporize at the contact position between the horn and workpiece, thereby affecting the weld quality of the joint [24]. Therefore, there is an urgent need to develop a method that can effectively improve the contact condition of the welding surface.

In production, when the warped upper sheet is placed upon lower sheet for joining, a gap is usually presented between the sheets. The gap between the workpieces can result in the poor contact condition, which is detrimental to the ultrasonic propagation during UW and weld formation [17,22,23]. At this context, a preload welding method is proposed to improve the contact condition between upper and lower workpieces. The influence of preloading on the joint strength, microstructure and stress and temperature distribution during UW process is investigated systematically. The ultrasonic wave transmission behaviors of UWed joints are also analyzed. This study offers a new approach to eliminate the negative effect by imperfect contact between workpieces during UWed carbon fiber reinforced polyamide 6 composite.

## 2. Experimental Procedure

### 2.1. Materials and Specimen Preparation

**Figure 1:**
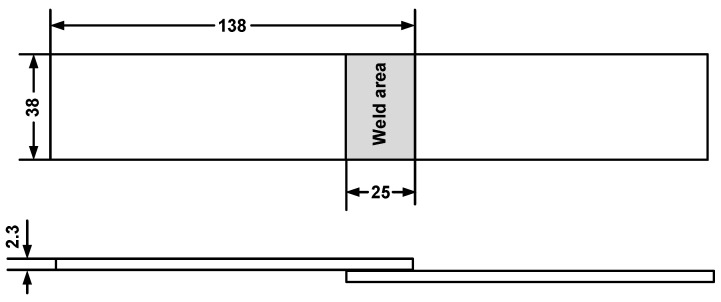
Schematic of the single-lap weld specimen (Dimensions in mm).

Carbon-fiber-reinforced nylon 6 composite with 30 wt.% short carbon fiber (C_f_/PA6, Tianfu Co., Ltd., Shanghai, China) was selected as test material, and the specimens with dimensions of 132.0 × 38.0 × 2.3 mm were prepared by a twin-screw extruder with two separate inlets injection molding. The specimens were stored in a barrel with desiccant, and the coupons were dried in an oven at 80 °C for 24 h before welding. Mechanical properties of the molded composite are shown in Table 1. The specimens were lapped by the upper and lower workpieces with an overlap distance of 25 mm as shown in Figure 1.

### 2.2. Ultrasonic Welding Process

**Figure 2:**
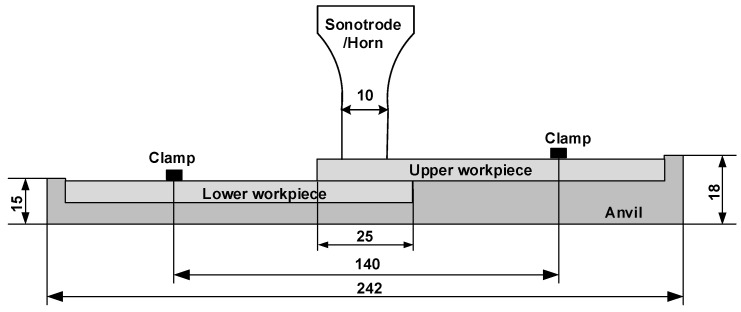
Schematic of ultrasonic welding of injection-molded 2.3-mm-thick lapped C_f_/PA6 composite without energy director (Dimensions in mm).

The UW process was performed using a KZH-2026 multifunction UW machine (Kaizheng Ultrasonic Technologies Co. Ltd., Weihai, China) with a nominal power of 2.6 kW, nominal frequency of 20 kHz, and nominal amplitude of 25 μm. The machine has three welding modes and time mode was used in this study. The welding process was controlled by presetting the delay time, welding time, and holding time. Then, the workpieces were welded at the nominal power of the machine. When the welding time (i.e., oscillation time) reached the preset value, the ultrasonic wave oscillation was stopped. The coupons were fixed using clamps to avoid their movement during welding (Figure 2). All specimens were welded by a 7075 aluminum horn with diameter of 10 mm.

### 2.3. Experimental Setup

**Figure 3:**
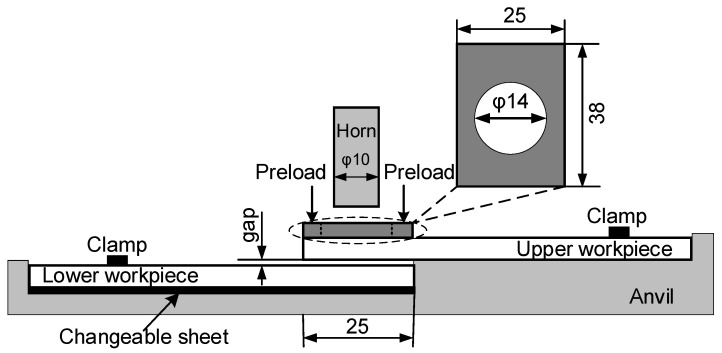
Schematic of ultrasonic welding with preload and gap between upper and lower workpieces (Dimensions in mm).

A preload was uniformly applied to overlapping area of the two workpieces (Figure 3). A controller was used to adjust the output voltage of the servo motor to control preload. The gaps between the upper and lower workpieces were adjusted by changing the thickness of a changeable sheet (Figure 3). Four different gaps, 0.5, 1.0, 1.5, and 2.0 mm, were utilized.

### 2.4. Mechanical Evaluation of the Welded Joints

**Figure 4:**
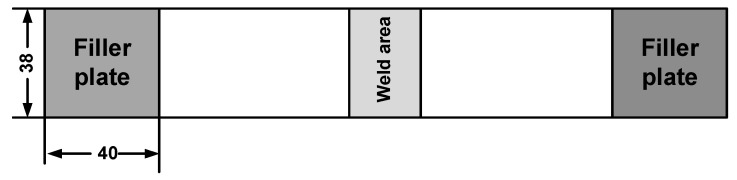
Schematic of single shear lap weld specimen (Dimension in mm).

Joint strength was evaluated by the peak load obtained from quasi-static tests, which performed by loading the welding specimen to failure in an MTS 810 tensile tester (ASTM D1002-2001) [25]. To minimize the bending stresses inherent during the testing of single-lap welding specimens, filler plates were attached to both ends of the specimen using masking tape to accommodate any sample offset (Figure 4). Load versus displacement curves were obtained during loading of the specimens at a stroke rate of 2 mm/min. Five replicates were performed per weld condition and the average weld strength were reported.

### 2.5. Finite Element Analysis

In this study, numerical simulations of the stress and deformation of single-lapped UWed joint with a gap between workpieces were carried out using the Finite Element Analysis (FEA) code, ANSYS. The ANSYS Workbench platform enables one to perform geometric modeling, material property definitions, meshing, and visualization.

In order to prevent the horizontal sliding of workpieces, the two workpieces with a large gap were fixed on the anvil by clamps in the experiment. In numerical routine, the samples displacements were blocked, and the lap zone of the upper workpiece was set to large displacement. And the contact conditions between the workpieces were defined as friction contact. For simplification, carbon fiber/polyamide 6 composite with 30 wt% fiber was considered to be an isotropic and linear elastic material, which was mainly due to the homogenous carbon fiber distribution in the matrix.

## 3. Result and Discussion

### 3.1. Effect of Preload on the Quality of Normal UWed Joints

**Figure 5:**
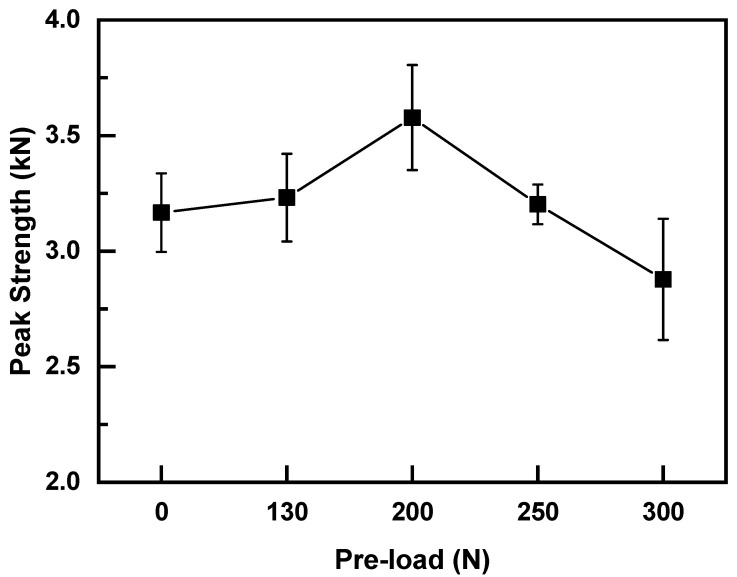
Effect of the preload force on strength of normal UWed joints (Welding pressure of 0.1 MPa and a welding time of 2.5 s).

**Figure 6:**
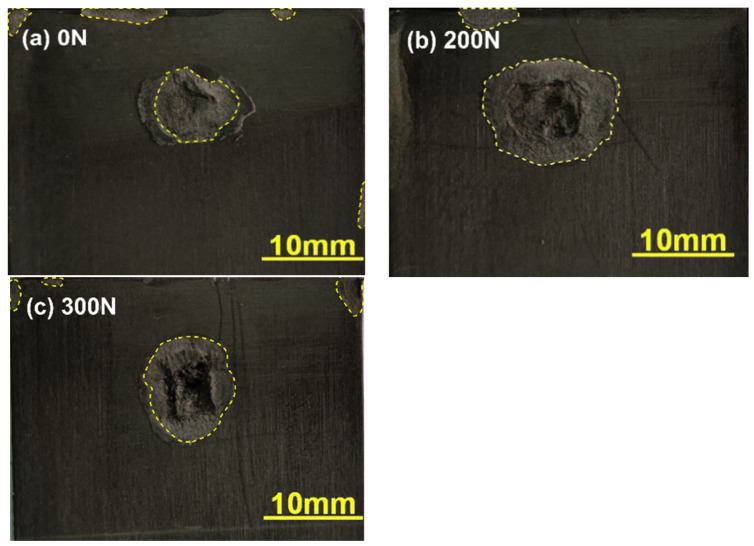
Effect of preload on nugget size of the joint.

To investigate the effect of preload on weld quality of normal UWed joint (i.e., joint without gap between upper and lower workpieces), various preload values (0, 130, 200, 250, and 300 N) were applied to the overlapping zone prior to UW. The welding tests were carried out with a welding pressure of 0.1 MPa, welding time of 2.5 s, and holding time of 3 s. Figure 5 presents the relation between joint strength and preload force. Strength of the joint increased initially and then decreased with the increasing preload. Joint welded with a preload of 200 N exhibited the maximum strength of 3.58 kN, which was 12.3% higher than that of UWed joint without preload (i.e., 3.16 kN). To further analyze the effect of preload on joint strength, welded surfaces of the joints were examined, and the results are shown in Figure 6. The nugget size of the joint prepared under preloading was significantly larger than that without preload. The weld size of the joint prepared under a preload of 200 N was the largest, implying the increase in joint strength by preloading is closely related to the increase in weld size.

**Figure 7:**
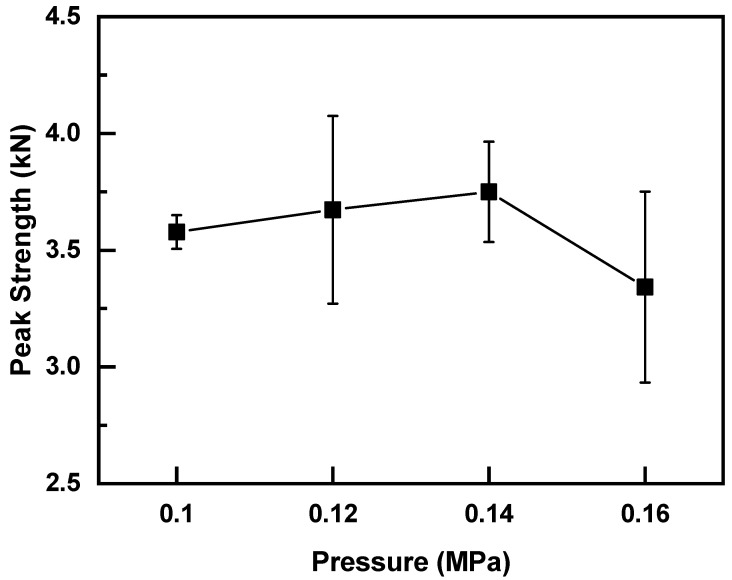
Effect of welding pressure on the joint strength (Preload of 200 N and welding time of 2.5 s).

Experimental results show that the preload can significantly increase the nugget area, and the nugget size affects the strength of the welded joints. Levy found that the heat generation at the initial stage of UW of polymer materials is dominated by Coulomb friction [26]. After the faying interface temperature reaches the glass temperature, viscoelastic dissipation becomes the main heat resource [27]. The protrusion deformation and contact area at the faying surface increase under the application of preload and welding pressure, which enhances the heat generation comes from Coulomb friction at the initial stage of UW. As a result, the joint enters into the viscoelastic heat generation stage earlier than that without preload. The temperature at the faying surface increases, the flowing and spreading speed of the melting layer accelerates, and the melted area increases, thereby improving the joint strength. However, higher preloads and welding pressures compact the bonding surface between upper and lower workpieces, which damps the ultrasonic oscillation, and the vibration amplitude decreases accordingly. This phenomenon is not conducive to the surface frictional heat generation, reducing heat accumulation at the welding surface and amount of melted material at the faying surface, which leads to the decrease in weld size and joint strength. Therefore, the welding pressure under the application of preload should be optimized. Various welding pressures (i.e., 0.10, 0.12, 0.14, and 0.16 MPa) were applied to the joint under preloading of 200 N. The welding time was maintained at 2.5 s. Figure 7 describes the effect of welding pressure on joint strength and the results are shown in Figure 7. Similar to the effect of preloading, the joint strength increased initially and then decreased with increasing welding pressure. The joint with welding pressure of 0.14 MPa showed the maximum strength of 3.75 kN, which was 18.7% higher than that of the weld without preload. Therefore, the optimal welding parameters were a preload of 200 N, welding pressure of 0.14 MPa, and welding time of 2.5 s.

### 3.2. Effect of Preload on Quality of UWed Joints with Poor Contact

**Figure 8:**
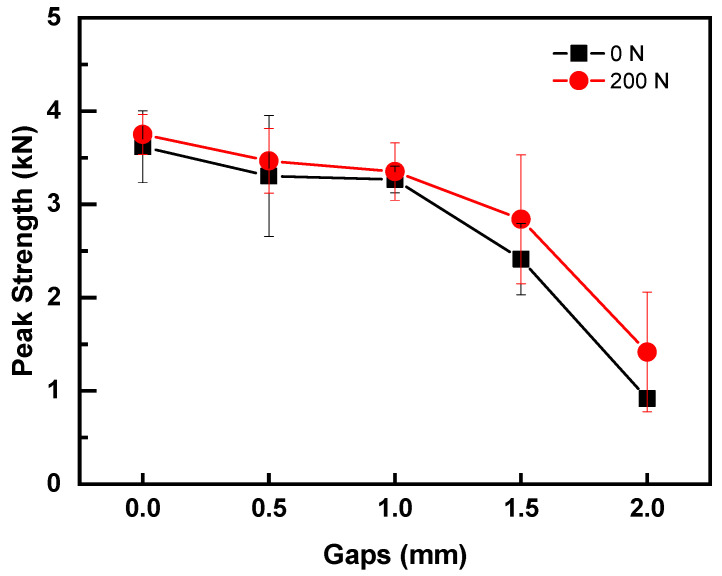
Effect of gap between workpieces on the strengths of normal and preloaded joints (200 N).

To investigate the effect of preload on welding joints with poor contact, the optimal welding parameters were adopted. Various gaps between upper and lower workpieces (i.e., 0, 0.5, 1.0, 1.5, and 2.0 mm) were introduced into normal and preloading joints (i.e., 0 N and 200 N). Figure 8 presents the effect of gap between the upper and lower workpieces. Overall, the joint strength decreased with increasing gap, regardless of the application of preload. Strengths of the joints with preload of 200 N were higher than that of the normal joints with the same gap, indicating that preload is beneficial to the strength of poorly contacted workpieces. Referring to Figure 8, the joint strength was affected slightly when the gaps between workpieces is in the range of 0~1.0 mm. The strengths of the normal and preloaded joints decreased by 6% and 10% for joints with 1.0 mm gap between workpieces. Further increasing the gap to 1.5 mm, the joint strength decreased to 2.41 kN and 2.84 kN, which were 66.7% and 75.7% of that normal joint, respectively. For the joints with a gap of 2.0 mm, the strengths of normal and preloaded joints dropped to 0.92 kN and 1.42 kN, respectively. The aforementioned results indicated that the existence of gaps between workpieces had a significant influence on the joint strength and the utilization of the preload can repair the strength of the joint with gap. However, the joint strength was still low when gaps between the upper and lower workpieces were above 2.0 mm, and the welding process needs to be further optimized.

**Figure 9:**
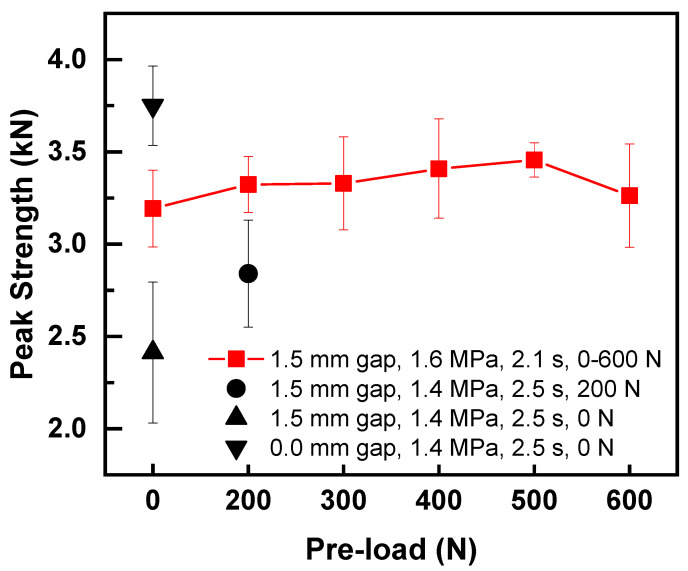
Effect of preload on the strength of joint with a gap of 1.5 mm.

Figure 9 shows the effect of preload on the strengths of poor contact joints (1.5 mm gap) welded with the optimized welding parameters (i.e., welding pressure of 0.16 MPa and welding time of 2.1 s). As shown, the strength of joint without preloading increased by 32.35% from 2.41 kN to 3.19 kN with the optimal parameters, which recovered to 88.3% of the normal joint. These findings showed that optimizing the welding parameters can improve the weld quality of joints with a gap of 1.5 mm between upper and lower workpieces. The applied preload recovered the joint strength to above 91.8% comparing to the normal joint. The joint with 500 N preload exhibited the highest peak load of 3.46 kN, which was 95.5% of that normal joint. The harmful influence caused by the introduction of gap between workpieces was mainly eliminated. These results showed that increasing the welding pressure and preload can amend the strength of the joint with poor contact between workpieces.

**Figure 10:**
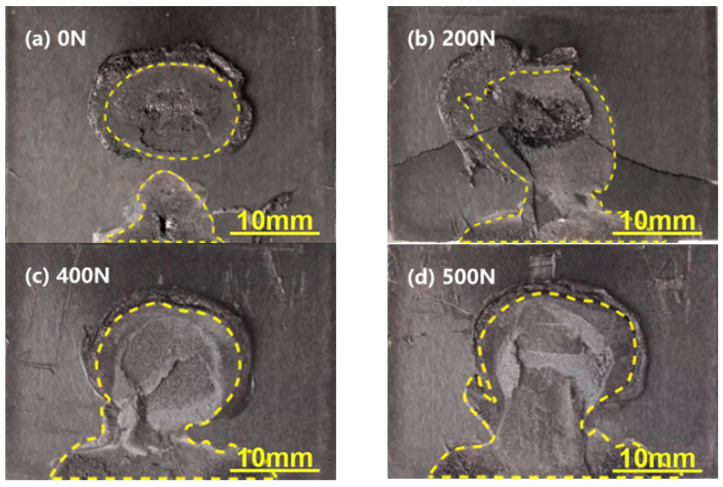
Effect of preload on the weld nugget size of joints with a gap of 1.5 mm.

To investigate the repairing mechanism of the application of preload, the fracture surfaces of joints under various preloads were examined as shown in Figure 10. Weld area of joints with preloads of 0, 200, 400, and 500 N were 167.8 mm^2^, 269.3 mm^2^, 309.8 mm^2^, and 350.5 mm^2^, respectively. The weld area increased with increasing preload, which implies the preload can improve the contact between the upper and lower workpieces for joint with a gap of 1.5 mm and enhance the wave energy transmission and absorption. It is concluded that the enhanced joint strength by preloading is mainly attributed to the increase in fusion area of the joint at the faying surface of C_f_/PA6 composite.

## 4. Improvement Mechanism of Preloaded Joints with Poor Contact

### 4.1. Stress and Deformation Analysis

To study the improving mechanism of discrepant joint by preloading, the stress and deformation of single-lapped UWed joint with a gap of 1.5 mm between workpieces were simulated. The contact conditions between the workpieces with welding pressure and with static pressure and preload were simulated with the commercial software Ansys EM20.1. The contact condition at the faying interface is mainly determined by the stress and deformation of the upper workpiece under the welding pressure and preload. Therefore, the stress distribution and deformation of the upper workpiece were simulated and analyzed under the application of welding pressure and preload.

**Figure 11:**
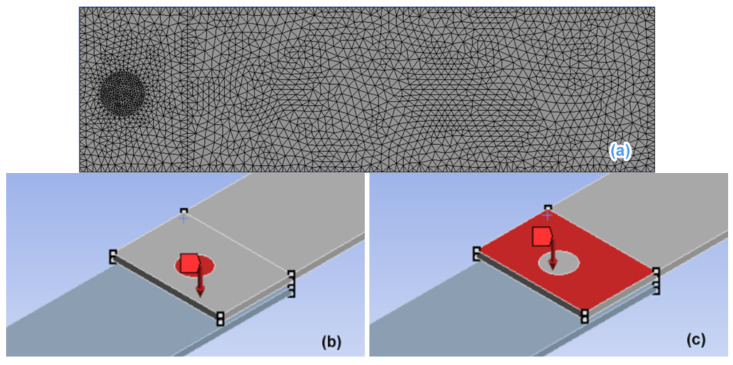
Pressure diagram of the lap region. (**a**) Mesh division of upper welding workpiece, (**b**) static pressure area, and (**c**) preload area.

The upper workpiece (dimensions: 132.0 × 38.0 × 2.3 mm) was meshed with a size of 1 mm, and the circular area at the overlap region was meshed with local mesh refinement (0.5 mm), as shown in Figure 11a. The overlap region of two workpieces was 25.0 mm × 38.0 mm, the gap between the upper and lower workpieces was 1.5 mm, the welding pressure was 0.16 MPa, the preload was 500 N, and the horn diameter was 10 mm. The static pressure was assumed to act on the 10-mm diameter area, which located in the center of upper workpiece, and the preload acts on rest of the lap area on the upper workpiece, as shown in Figure 11b and Figure 11c, respectively.

The upper workpiece was fixed constraint except the overlapped region. The stresses under welding pressure (*F*) and preload (*P*) can be obtained by Equations (1) and (2):

(1) $$\sigma =\frac{Fl}{2W}\;$$

(2) $$\sigma_{0}=\frac{Pl^{2}}{2W}$$

where *l* = 25 mm is the length of the overlapped region and *W* is section modulus in bending. Then, the strain, ε, is calculated by the following:

(3) $$\sigma +\sigma_{0}=C:\varepsilon \;$$

where *C* is the elastic matrix. The deformation of the overlapped region in upper workpiece (i.e., displacement), u, can be deduced by Equation (4):

(4) $$\tilde{\varepsilon }=\frac{1}{2}\left[{\left(\nabla \vec{u}\right)}^{T}+\nabla \vec{u}\right]\;$$

**Figure 12:**
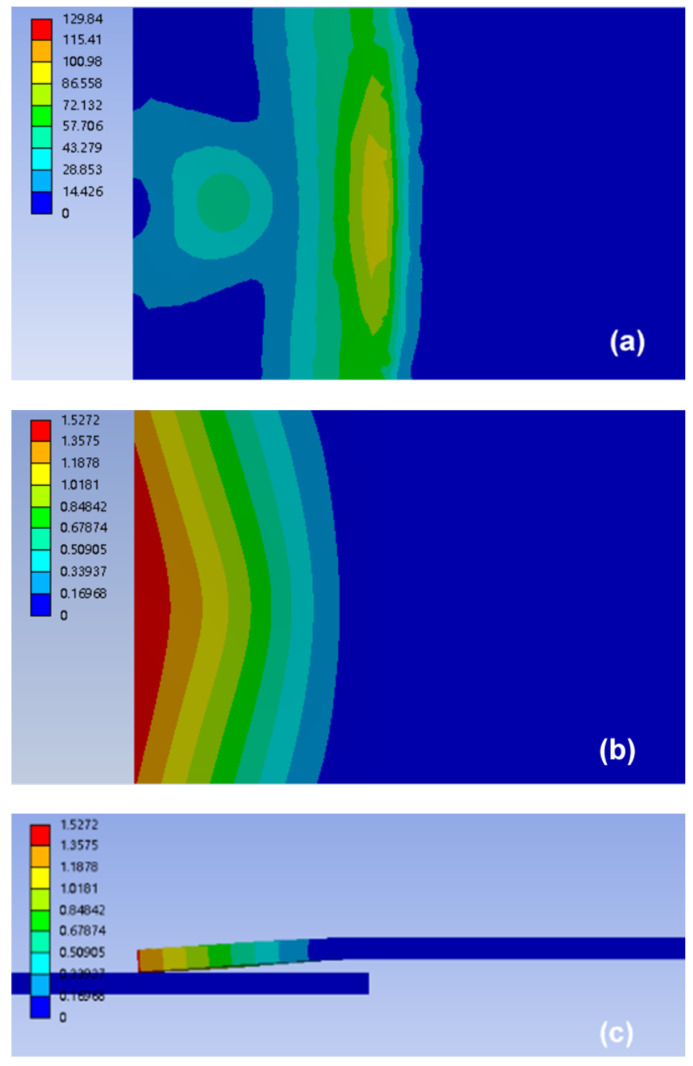
Simulation of stress and deformation of the upper workpiece for joint with gap of 1.5 mm under static pressure: (**a**) stress distribution of upper workpiece (Units in MPa), (**b**) top view, and (**c**) side view of the deformation distribution of the upper workpiece (Units in mm).

**Figure 13:**
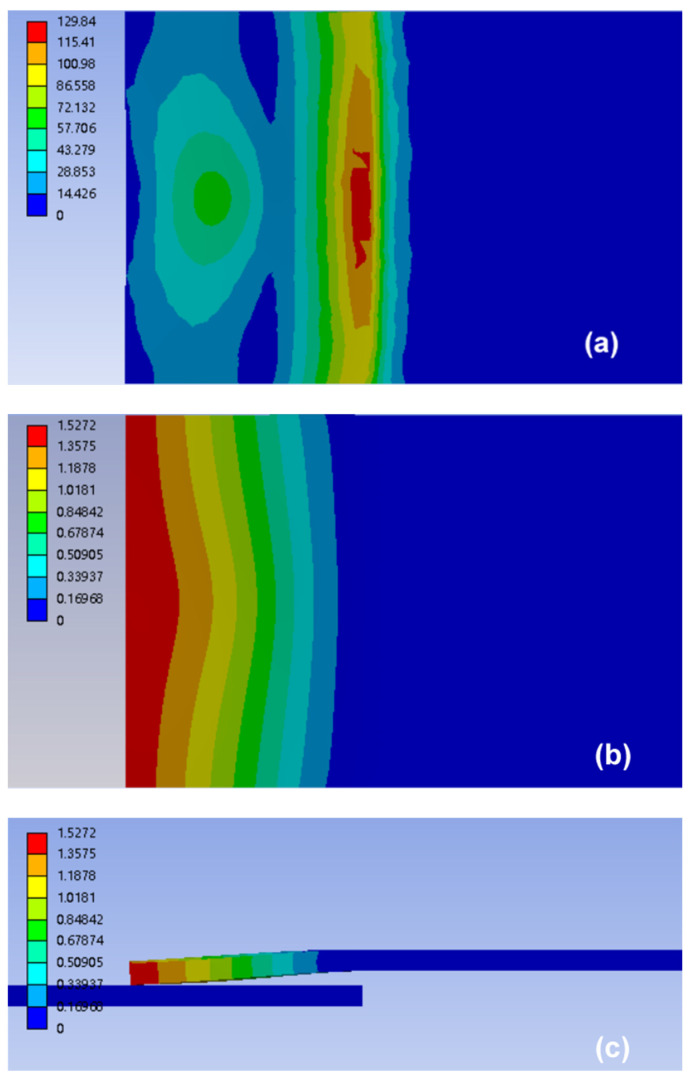
Simulation of stress and deformation of the upper workpiece for joint with gap of 1.5 mm under static pressure and preload: (**a**) stress distribution of the upper workpiece (Units in MPa), (**b**) top view, and (**c**) side view of the deformation distribution of the upper workpiece (Units in mm).

The stresses and deformations of the upper workpiece under merely static pressure and combination of preload and static pressure are shown in Figure 12 and Figure 13, respectively. The simulated results showed that the stress and deformation of the upper workpiece under preload and static pressure was greater than that of the workpiece under static pressure; the high deformation area was also larger (see Figure 12a,b and Figure 13a,b). The upper workpiece formed a cantilever beam due to the existence of gap. Thus, stress of the upper workpiece was mainly concentrated in the support end of the anvil and the center of the lap area. This condition resulted in the maximum deformation at end of the upper workpiece (Figure 12c and Figure 13c), forming an approximate wedge gap, which gradually worsened the contact condition between the upper and lower workpieces. However, the stress of the upper workpiece was larger than that without preload due to the combined effect of preload and static pressure. In addition, the downward deformation (i.e., Figure 13c) was larger than that of static pressure (i.e., Figure 12c). Thus, the gap between workpieces was narrowed, and the contact condition of the upper and lower workpieces was improved under preload.

### 4.2. Modeling of Ultrasonic Transmission with Preloading

The high-frequency ultrasonic vibration transfers to the workpieces through welding horn during UW of thermoplastic composites. Ultrasonic waves are reflected, transmitted, and absorbed in the form of mechanical waves at the faying interface and inside the material [16,27]. Under the application of welding pressure, the heat generations by friction at the faying interface and viscoelastic dissipation of the material itself are concentrated at the welding interface to enable the material to melt, outflow, solidify, and form a welded joint. According to the analysis in Section 4.1, the preload improves the contact condition between upper and lower workpieces, thereby enhancing the transmission and absorption of ultrasonic dissipation between the workpieces.

**Figure 14:**
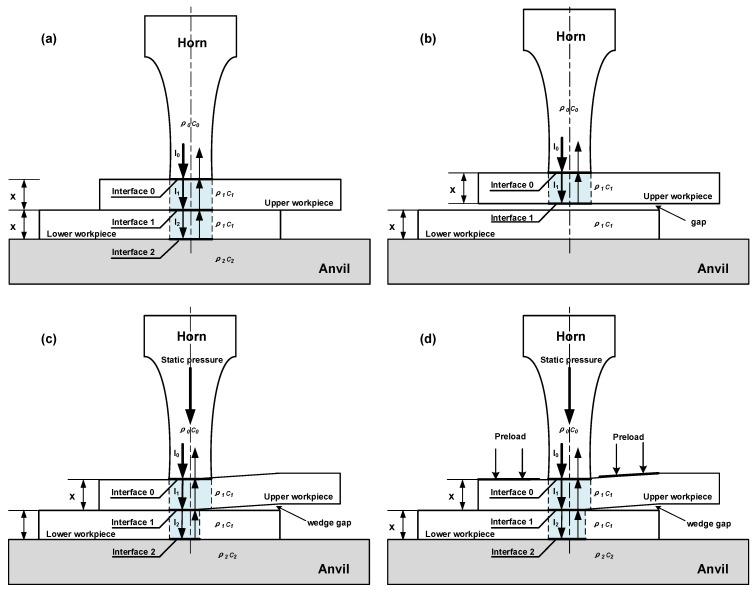
Schematic of the ultrasonic wave propagation during UW: (**a**) perfect contact, (**b**) with gap between workpieces, and with a wedge gap under (**c**) static pressure, or (**d**) static pressure and preload.

Khmelev et al. constructed an energy dissipation model for ultrasonic welded thermoplastic joints, which provides a basis for the analysis of ultrasonic energy transfer [16]. In the model, the workpieces are regarded as homogeneous materials, assuming that the upper and lower parts are perfect in contact, and the contacted area is the same as that of the horn diameter. The influence of the welding pressure on the contact condition between parts is not considered. In our previous research [28], a quality evaluation model of C_f_/PA66 composite joints is proposed based on the Khmelev ultrasonic energy absorption model. In this study, the quality evaluation model is used to analyze the effect of welding pressure and preloading on the contact condition between upper and lower workpieces and ultrasonic energy transmission and absorption. Figure 14 presents the schematic of ultrasonic propagation.

Figure 14a shows that ultrasonic vibrations with an intensity of *I*_0_ are initially generated from the ultrasonic oscillation system with an acoustical impedance of *z*_0_ = *ρ*_0_*c*_0_. Part of the ultrasonic waves is reflected back to the horn, and part of the waves passes through the boundary. Thus, the coefficient of reflection $\eta_{i}$ and wave transmission factor $d_{i}$ at the interface can be determined using Equations (5) and (6) [28]. Interface 0 is the surface between the horn and upper workpiece, interface 1 is the upper and lower workpiece interface, and interface 2 is the lower workpiece and anvil surface.

(5) $$\eta_{i}={\left(\frac{\rho_{u}c_{u}-\rho_{l}c_{l}}{\rho_{u}c_{u}+\rho_{l}c_{l}}\right)}^{2}$$

(6) $$d_{i}=1-\eta_{i}$$

where i=0,1,2, $\rho_{u}$ and $\rho_{l}$ are the density of the upper and lower adherends, $c_{u}$ and $c_{l}$ are the sound velocities in the adherends.

For an incident wave with energy intensity I, the wave reflection or transmission at the faying surface are calculated using Equation (7) and Equation (8), respectively.

(7) $$I_{ir}=\eta_{i}I\;$$

(8) $$I_{it}=d_{i}I$$

Then, the wave intensity of the upper workpiece (i.e., C_f_/PA6 composite) is expressed as following:

(9) $$I_{1}=I_{0}d_{0}=I_{0}\left[1-{\left(\frac{\rho_{0}c_{0}-\rho_{1}c_{1}}{\rho_{0}c_{0}+\rho_{1}c_{1}}\right)}^{2}\right]\;$$

Ultrasonic wave would attenuate during transmission, and it attenuates to $I_{1}e^{-\alpha x}$ as the wave passes through the thermoplastic composite workpiece with a thickness of x and attenuation coefficient α. The energy dissipation in upper workpiece is calculated using Equation (10).

(10) $$W_{i1}=(I_{1}-I_{1}e^{-\alpha x})S+(\eta_{2}I_{1}e^{-3\alpha x}-\eta_{2}I_{1}e^{-4\alpha x})S_{1}\;$$

where $S_{1}$ is area of the welding zone (i.e., area of interface 1), and $\eta_{2}$ is the wave reflection coefficient of the interface. $S_{2}$ is the area of interface 2, and the energy absorbed by the lower workpiece is defined as *W*_*i*2_:

(11) $$W_{i2}=(I_{1}e^{-\alpha x}-I_{1}e^{-2\alpha x})S_{1}+(\eta_{2}I_{1}e^{-2\alpha x}-\eta_{2}I_{1}e^{-3\alpha x})S_{2}\;$$

If a gap exists between upper and lower workpieces, then the reflection coefficient of the interface between C_f_/PA6 and air reaches nearly 0.99 [22], indicating that almost all of the ultrasonic waves are reflected by the interface, and ultrasonic welding fails, as shown in Figure 14b.

The simulation results of stress and deformation in Section 4.1 show that when the gap between upper and lower workpieces is above 1.5 mm, a wedge gap would form between the workpieces under the application of welding pressure. The area for ultrasonic transmission at interface *I* becomes $S_{1w}$ and the corresponding reflection area at interface *II* is $S_{2w}$, as shown in Figure 14c. Similarly, under the application of welding pressure and preload, the transmission area at interface *I* is $S_{1p}$($S_{1p}>S_{1w}$), and the reflection area at interface *II* is $S_{2p}$, as shown in Figure 14d. As seen in Figure 14c,d, the transmission area at interface *I* equals to the reflection area at interface *II*, namely, $S_{1w}$ = $S_{2w}$, $S_{1p}$ = $S_{2p}$.

Comparing to the joint under welding pressure, the absorbed energies by upper and lower workpieces, $\Delta W_{i1}$ (Equation (12)) and $\Delta W_{i2}$ (Equation (13)), are lower than that with application of welding pressure and preload once the gap between the upper and lower workpieces is above 1.5 mm.

(12) $$\Delta W_{i1}=(I_{1}\eta_{2}e^{-3\alpha x}-I_{1}\eta_{2}e^{-4\alpha x})(S_{1p}-S_{1w})\;$$

$$\Delta W_{i2}=(I_{1}e^{-\alpha x}-I_{1}e^{-2\alpha x})(S_{1p}-S_{1w})+(I_{1}\eta_{2}e^{-2\alpha x}-I_{1}\eta_{2}e^{-3\alpha x})(S_{2p}-S_{2w})$$

(13) $$\Delta W_{i2}=(I_{1}e^{-\alpha x}-I_{1}e^{-2\alpha x}+I_{1}\eta_{2}e^{-2\alpha x}-I_{1}\eta_{2}e^{-3\alpha x})(S_{1p}-S_{1w})\;$$

The whole energy dissipation during UW process can be expressed as:

(14) $$\Delta W_{i}=W_{i1}+\Delta W_{i1}+W_{i2}+\Delta W_{i2}\;$$

These analyses show that the energy absorbed by the upper and lower workpieces during UW with preloading is higher than that of normal joint. Thus, the viscoelastic dissipations in workpieces increase as well.

Once the stress, deformation and ultrasonic dissipation during UW are investigated, the heat generations at the faying interface for joints made with a welding pressure of 0.16 MPa, a welding time of 2.1 s, a gap of 1.5 mm and various preload (i.e., 0 N, 200 N, 400 N, 500 N), are simulated with the commercial software Ansys EM20.1 (the frictional heat generation between workpieces is not considered for simplicity).

A strain energy in the thermoplastic composites is generated due to viscosity of the polymer. Therefore, the viscoelastic dissipation (i.e., the ultrasonic vibration energy absorbed by the polymer material) equals to the strain energy in a single cycle. The temperature rise (Δ*T*) can be calculated from the energy dissipation in polymer materials in a single cycle, as follows [29]:

(15) $$\Delta T=\frac{\Delta W_{i}}{c\rho }\;$$

where c = 1.60 kJ/kg·K and ρ = 1130 kg/m^3^ are specific heat capacity and density of C_f_/PA6, respectively. In addition, the thermal conductivity of C_f_/PA6 is λ = 0.31 W/m·K.

The melting material is generated only on the faying surface between upper and lower workpieces during UW process, and it is very thin. In addition, the welding time is short (less than 3 s) and thermal conductivity of C_f_/PA6 is poor. Therefore, the contacts between welding parts and air, and fixture and fixture are regarded as insulation condition [30]. The heat transfer is simplified as a two-dimensional heat conduction along the faying surface.

(16) $$\lambda \left(\frac{\partial^{2}T}{\partial x^{2}}+\frac{\partial^{2}T}{\partial y^{2}}\right)+\Delta W_{i}=\rho c\frac{\partial T}{\partial t}$$

where T is temperature of a specific position, x and y is coordinate of the temperature field, t = 2.1 s is welding time.

**Figure 15:**
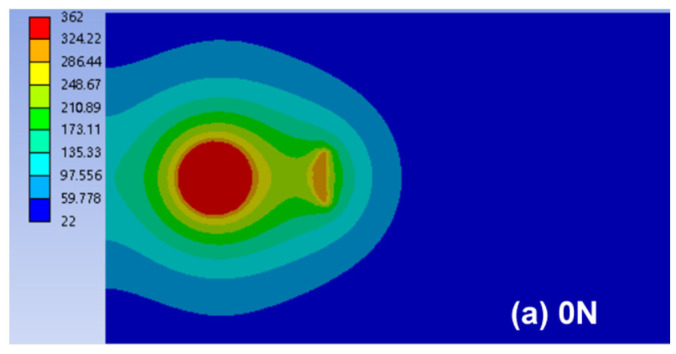

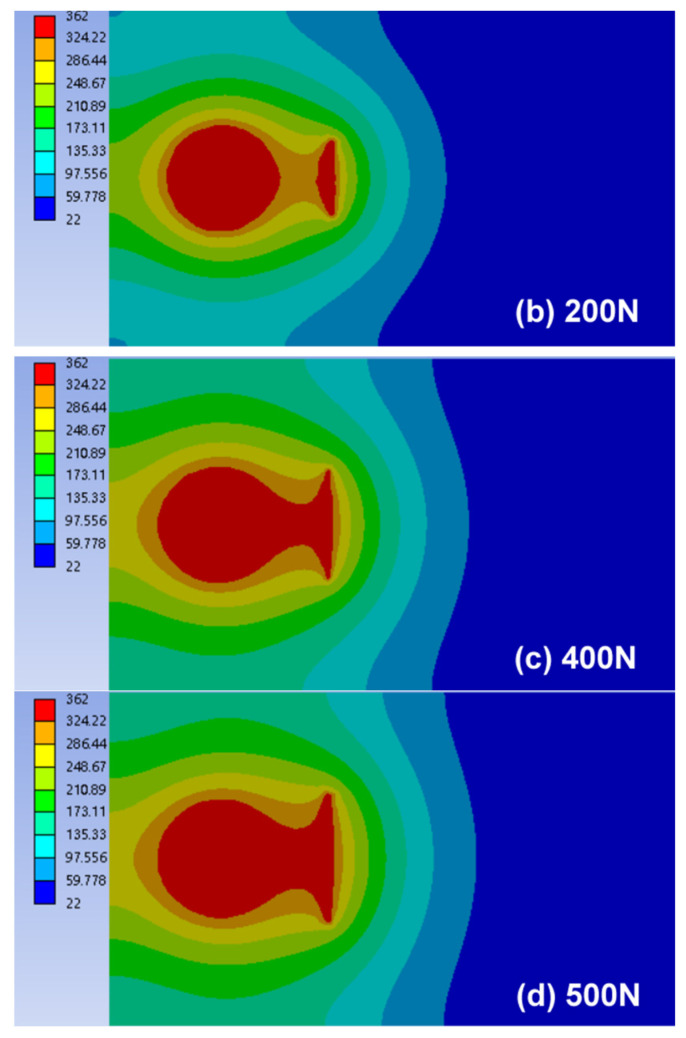
Simulation results of the temperature distribution for joint with 1.5 mm gap between workpieces under various preloads (Units in °C).

Figure 15 shows the temperature distribution of joint welded with 2.1 s at room temperature (22 °C). As shown, the maximum temperature distribution expanded at the faying interface gradually with increasing preload, which was consistent with the varying trends in the joint strength (Figure 9) and nugget area (Figure 10). Combining the results of nugget size, stress and deformation, and ultrasonic dissipation during UW process, it was concluded that the application of preload improved the contact condition between workpieces and increased the transmission of ultrasonic vibration; therefore, the nugget size and joint strength increased under preload.

## 5. Conclusions

Extensive UW tests of 2.3-mm-thick nylon6 composites with 30 wt% carbon fiber were conducted to investigate the effect of preload on weld quality. The results showed that under the given welding parameters, the preload can greatly improve the joint strength, especially in the case of a gap between workpieces. Therefore, the application of a preload is recommended during UW of composite materials in practical manufacturing applications. However, the welding parameters and magnitude of the preload should be optimized experimentally to ensure effective joining with different materials, plate thicknesses, and types of UW machines. The application of preload should be further studied to solve problems, such as excessive indentation, thinning of penetration, excessive extrusion of solute, plate deformation, and cracking at the edge of plates caused by excessive welding pressure. The main conclusions are as follows:

(1) Preload can improve the weld quality of the composites, and the strength increased by 18.7% for the normal joint with preload of 200 N made with optimal welding parameters;
(2) The existence of gap between upper and lower workpieces had a considerable negative influence on joint strength. When the gap was smaller than 1.0 mm, the joint strength decreased slightly while to 66.7% and 25.4% of the normal joint for gaps of 1.5 mm and 2.0 mm.
(3) Preload can repair the strengths of joints with gaps. The strength of the joint with 1.5 mm gap can be recovered to 95.5% of the normal joint under optimized welding parameters;
(4) Application of preloading improved the strength of the joint with poor contact, which was mainly because the preload compacted the contact between workpieces and increased the ultrasonic transmission, resulting in the increase in nugget area.

## Figures and Tables

**Table 1:** Mechanical properties of molded 2.3-mm-thick C_f_/Nylon 6 composite coupons.

	Tensile Strength (MPa)	Elastic Strength (MPa)	Poisson’s Ratio	Density (kg/m^3^)
Nylon6	74	2501	0.34	1130
C_f_/PA6	89.2	7532	0.34	1260

## Data Availability

The data presented in this study are available on request from the corresponding author.
